# EXPERIENCES OF VETERANS, CAREGIVERS, AND VA HOME-BASED CARE PROVIDERS POST-HURRICANE IAN

**DOI:** 10.1093/geroni/igad104.3729

**Published:** 2023-12-21

**Authors:** Leah Haverhals, Chelsea Manheim, Deisy Hernandez-Lujan

**Affiliations:** Department of Veterans Affairs Rocky Mountain Regional VA Medical Center, Aurora, Colorado, United States; Department of Veterans Affairs Rocky Mountain Regional VA Medical Center, Aurora, Colorado, United States; Department of Veterans Affairs Rocky Mountain Regional VA Medical Center, Aurora, Colorado, United States

## Abstract

On September 28, 2022, Hurricane Ian pummeled parts of south Florida. Disaster and climate change research has shown that disasters exacerbate inequalities, especially amongst older and physically vulnerable people. Florida has a large population of Veterans managing multiple chronic health conditions and receiving long-term services and supports in-home. Many such Veterans receive care from the Veterans Health Administration (VA) Home Based Primary Care (HBPC) or Medical Foster Home (MFH) programs. To describe how VA staff provided high quality care during and post-hurricane, and how Veterans and caregivers accessed needed healthcare and supports post-hurricane, we conducted a site visit to the Fort Myers, Florida area in May 2023. We conducted N=25 in-person interviews with VA HBPC and MFH staff, Veterans, and family and VA MFH caregivers. Findings from qualitative thematic analysis showed that Veterans and caregivers felt highly supported by VA care teams who made proactive changes in the days prior to and the months post-hurricane. Staff efforts post-hurricane focused on improving care coordination in anticipation of future disasters, especially around communicating with Veterans and their caregivers, and a VA workgroup formed to implement changes. Some Veterans and caregivers experienced significant challenges during and post-hurricane, with a few displaced and having to relocate, and others having difficulty accessing needed oxygen and suffering injuries related to the hurricane. As climate change causes more severe natural hazard events, lessons learned from this project can inform how to better support healthcare staff, older adults, and their caregivers pre-and post-major disasters.

